# Multimodal Metaverse Healthcare: A Collaborative Representation and Adaptive Fusion Approach for Generative Artificial-Intelligence-Driven Diagnosis

**DOI:** 10.34133/research.0616

**Published:** 2025-03-12

**Authors:** Jianhui Lv, Adam Slowik, Shalli Rani, Byung-Gyu Kim, Chien-Ming Chen, Saru Kumari, Keqin Li, Xiaohong Lyu, Huamao Jiang

**Affiliations:** ^1^ The First Affiliated Hospital of Jinzhou Medical University, Jinzhou 121012, China.; ^2^ Koszalin University of Technology, Koszalin 98701, Poland.; ^3^ Chitkara University, Rajpura, Punjab 140401, India.; ^4^ Sookmyung Women’s University, Seoul, Republic of Korea.; ^5^ Nanjing University of Information Science & Technology, Nanjing, China.; ^6^ Chaudhary Charan Singh University, Meerut, India.; ^7^ State University of New York, New Paltz, NY 12561, USA.

## Abstract

The metaverse enables immersive virtual healthcare environments, presenting opportunities for enhanced care delivery. A key challenge lies in effectively combining multimodal healthcare data and generative artificial intelligence abilities within metaverse-based healthcare applications, which is a problem that needs to be addressed. This paper proposes a novel multimodal learning framework for metaverse healthcare, MMLMH, based on collaborative intra- and intersample representation and adaptive fusion. Our framework introduces a collaborative representation learning approach that captures shared and modality-specific features across text, audio, and visual health data. By combining modality-specific and shared encoders with carefully formulated intrasample and intersample collaboration mechanisms, MMLMH achieves superior feature representation for complex health assessments. The framework’s adaptive fusion approach, utilizing attention mechanisms and gated neural networks, demonstrates robust performance across varying noise levels and data quality conditions. Experiments on metaverse healthcare datasets demonstrate MMLMH’s superior performance over baseline methods across multiple evaluation metrics. Longitudinal studies and visualization further illustrate MMLMH’s adaptability to evolving virtual environments and balanced performance across diagnostic accuracy, patient–system interaction efficacy, and data integration complexity. The proposed framework has a unique advantage in that a similar level of performance is maintained across various patient populations and virtual avatars, which could lead to greater personalization of healthcare experiences in the metaverse. MMLMH’s successful functioning in such complicated circumstances suggests that it can combine and process information streams from several sources. They can be successfully utilized in next-generation healthcare delivery through virtual reality.

## Introduction

The metaverse is a collective virtual shared space created by the fusion of physically persistent virtual spaces and enhances physically persistent virtual spaces [1]. Today, the metaverse has emerged as an overarching platform with wide-reaching consequences in delivering healthcare services and educating health professionals. Although the metaverse has received considerable healthcare consideration, it has other applications, such as integrated energy systems [2] and digital enterprise management [3]. Consequently, this showcases the potential of the metaverse as a revolutionary platform. By fashioning interactive 3-dimensional (3D) spaces, the metaverse enhances remote patient management, virtual interaction, and teamwork in healthcare [4,5]. Medicine in virtual worlds presents unprecedented benefits, as practitioners can attend to patients, retrieve medical information, and collaborate with other professionals regardless of physical location. The possibilities are further extended by the infusion of generative artificial intelligence (AI) in metaverse healthcare environments, which permit realistic simulations, individualized treatment programs, and tailored educational pathways for patients and health workers alike [6]. When this digital transformation in healthcare is imminent, there is a growing need to formulate appropriate strategies to interrogate and interpret the various types of data generated in such complex electronic networks, which are now considered impossible [7].

As regards their technological aspect, multimodal machine learning is a unique model that is appealing in the healthcare of the metaverse, given that it applies multiple sources of patient data for better diagnosis and monitoring results [8–11]. Within the scope of healthcare in a metaverse, relevant modalities may include text and audio medical records, patient–doctor conversations, medical images and remote monitor data, virtual examinations, and even patient haptic data. This data type is paramount in defining the completeness of the patient’s profile in a virtual setting. It enables health practitioners to make practical decisions on the patient’s health in the virtual realm. Additionally, large language models and text-to-image generators as generative AI technologies involve adding theoretical patients with temporal information to real patients, and this kind of synthesized data had to be integrated with actual patient data [12–16]. This duality of multimodal data analysis and generative AI presents the possibility of greatly enhancing today’s practices of metaverse healthcare.

Multimodal representation learning and multimodal fusion are 2 core components of creating AI systems for metaverse healthcare [17,18]. Contemporary multimodal representation approaches often focus on intrasample collaboration, resulting in weak feature representation across patient cases. This shortcoming is particularly disadvantageous in metaverse healthcare, where patient records could be pooled from several virtual environments and under different circumstances. Also, the majority of available solutions provide care and attention to noisy data [19]. The same problems could also be found in reverse: the inverse variation of adjusting capabilities had caused many interference effects from or to the data from generative AI models during patient monitoring [20–22]. These challenges create substantial barriers to developing and deploying robust AI-based diagnostic and monitoring systems in metaverse healthcare environments [23,24]. This may harm patient care and safety in virtual clinics where such technologies are implemented.

In response to challenges, we propose a novel multimodal learning framework for metaverse healthcare, MMLMH. MMLMH is advanced by incorporating intra- and intersample collaborative representation learning as well as adaptive fusion techniques to tackle the heterogeneous and dynamic features of health data in virtual settings. The method provides a more holistic view of complex relations between health data and health phenomena across multiple modes, thereby improving the quality of features for AI-centric metaverse healthcare systems.

The primary advancement of MMLMH is its innovative method for processing healthcare data within virtual environments, characterized by 3 essential architectural innovations. Our collaborative intra- and intersample representation learning mechanism effectively addresses the challenge of maintaining clinical relevance in virtual consultations. In contrast to current approaches that handle each modality separately, MMLMH’s dual-level collaboration effectively captures immediate relationships within individual patient interactions and overarching patterns across multiple cases.

The second core innovation focuses on MMLMH’s adaptive fusion architecture, specifically tailored for the dynamic characteristics of healthcare interactions within the metaverse. This framework presents a calibration mechanism that continuously evaluates and modifies the reliability weights of various modalities in real time. This capability is essential for processing diverse quality levels of virtual avatar movements, transmitted physiological sounds, and patient–provider dialogues.

MMLMH’s integrated approach to managing real and synthetic healthcare data in virtual environments is a notable characteristic. The framework includes a specialized encoder that learns the relationships between physical clinical indicators and their virtual representations. This innovation facilitates the integration of AI-generated clinical scenarios with actual patient data, ensuring consistent performance across hybrid datasets.

## Results

### Setup

To assess how effective the proposed MMLMH framework is in the context of multimodal metaverse healthcare provision, we performed experiments on 2 datasets:

• Multimodal Corpus of Sentiment Intensity (CMU-MOSI) [25]: CMU-MOSI is a dataset of multimodal language focused on multimodal sentiment analysis, containing 2,199 video segments from 93 YouTube movie review videos. We applied the sentiment scoring method of the reviews to this dataset and augmented the data by the synthetic virtual environment to recreate virtual world healthcare consultations.
• Medical Information Mart for Intensive Care III (MIMIC-III) [26]: The MIMIC-III dataset is an extensive open-access database that includes health-related information of over 40,000 subjects who spent time in critical care units of the Beth Israel Deaconess Medical Center from 2001 to 2012, while their identities were anonymized.

Both datasets were modified to fit the requirements of utilizing an MMLMH framework that incorporates text, audio, and visual modalities in addition to synthetic metaverse environment features.

Implementing MMLMH in virtual healthcare environments necessitates allocating specific computational resources to guarantee real-time processing capabilities while maintaining diagnostic reliability. The implementation employed a distributed computing architecture with primary processing nodes outfitted with 8 NVIDIA A100 graphics processing units (40-GB video RAM) to facilitate the core multimodal fusion operations. The memory requirements exhibited a correlation with the complexity of virtual consultation. The optimal performance was achieved by utilizing 64 GB of system RAM, which facilitated managing concurrent patient interactions and maintaining the active feature cache. The storage specifications included a 2-TB nonvolatile memory express solid-state drive configuration, with 500 GB allocated for model weights and 1.5 TB for the temporary storage of data and the processing of features during virtual consultations.

The real-time performance metrics of the framework demonstrated processing latencies of 150 ms for standard virtual consultations involving the simultaneous transmission of text, audio, and visual data streams. To ensure the requisite level of performance, the network bandwidth must be at least 100 Mbps with sub-20-ms latency to maintain fluid virtual interactions. When subjected to a load of 50 concurrent virtual consultations, the system demonstrated stable performance while consuming approximately 85% of the available graphics processing unit computing capacity and 70% of the available memory resources. In distributed deployments, load balancing was implemented across multiple processing nodes, with each node handling up to 20 concurrent sessions while maintaining response times below 200 ms.

The optimization process reduced the initial model size from 380 to 125 GB by applying quantization and pruning techniques while maintaining diagnostic accuracy. The framework exhibited effective resource scaling, necessitating approximately 2 GB of supplementary video RAM for each concurrent virtual consultation while sustaining performance within the established clinical parameters. These specifications enabled MMLMH to consistently process complex multimodal health data streams within real-world virtual healthcare scenarios.

We compared the MMLMH framework with 6 baseline methods:

• Uni2Mul [27]: a conformer-based multimodal emotion classification model adapted for health status prediction in virtual environments
• CSID [28]: a multimodal image fusion algorithm modified to integrate virtual and real medical imaging data for enhanced clinical diagnosis in metaverse settings
• DAMUN [29]: a domain-adaptive human activity recognition network based on multimodal feature fusion, adapted for patient behavior analysis in virtual healthcare environments
• HAMF [30]: a hierarchical attention-based multimodal fusion framework utilizing imaging, genetic, and clinical data, extended to incorporate metaverse-specific features for early disease detection
• MFNet [31]: a multimodal fusion network for intensive care unit patient outcome prediction, modified to handle virtual patient data streams
• MD-RCNN [32]: a multimodal data-based recurrent convolutional neural network for disease risk prediction, adapted to process synthetic data from metaverse healthcare simulations

Selection criteria were on the methods that integrated different data types in the healthcare setting. Uni2Mul’s conformer-based architecture was selected based on its ability to perform temporal alignment of different modalities. This was relevant to aligning patients’ spoken words, movements, and clinical streams during virtual consults. Again, CSID enabled the fusion of complex images and provided methods for combining visual features with other modalities. This is a key function in rendering avatar interactions and medical images in the same context within metaverse situations.

DAMUN’s domain-adaptive approach provided a robust foundation for handling the heterogeneous nature of healthcare data, particularly in bridging the gap between physical and virtual clinical indicators. The effectiveness of HAMF’s hierarchical attention mechanism in selecting important features over several modalities was critical in dealing with complex virtual clinical presentations. Another more realistic viewpoint came from using MFNet, which showed during implementation in intensive care unit data that it was possible to work with clinical data streams in real time. Simultaneously, MD-RCNN, with its recurrent structure, demonstrated an ability to utilize temporal information from different healthcare-related data sources.

These methods were most notable as they represented substantial milestones in the evolution of the multimodal fusion frameworks, each having unique strengths in addressing various aspects of the multimodal integration problems. The selection of these methods made it possible to complete independent validation of the performance of MMLMH against the established approaches concerning the complex requirements of data fusion in metaverse healthcare.

In order to evaluate the performance of our MMLMH framework for each of the procedures and to the culmination of this study, as well as provide a rationale for the choice of evaluation measures, we adopted a set of evaluation metrics aligned with the nature of each dataset and the type of healthcare tasks performed within the metaverse.

We evaluated regression performance and health classification accuracy using standardized metrics [33]. For example, continuous health scores are typically evaluated using mean absolute error, which describes the average size of prediction errors. While predicting survival, results are subjected to a Pearson correlation coefficient, which measures the degree of relationship between the variable and observed outcomes. In quantifying the health residues, we used binary classification accuracy (Acc-2) to categorize the health states as either positive or negative sickness, the F1 score was calculated to combine precision and recall measurements, and the baseline 7-class rascal classifier (Acc-7) used demonstrated the health state for the metaverse.

We implemented the MMLMH framework using PyTorch. The hyperparameters were tuned using a grid search approach, optimizing for performance on the validation sets. Key parameter settings are presented in Table 1.

**Table 1.** Hyperparameter settings for the MMLMH framework

Parameter	Value
Maximum text sequence length ($l_{\tau }$)	512
Maximum audio sequence length ($l_{\alpha }$)	1,000
Maximum visual sequence length ($l_{\nu }$)	100
Text feature dimension ($F_{\tau }$)	768
Audio feature dimension ($F_{\alpha }$)	128
Visual feature dimension ($F_{\nu }$)	2,048
Intrasample loss weight ($\beta_{1}$)	0.7
Intersample loss weight ($\beta_{2}$)	0.8
Reconstruction loss weight ($\beta_{3}$)	0.5
CMD order (K)	5
Hidden layer size	256
Learning rate	1.00 × 10^−4^
L2 regularization	1.00 × 10^−5^
Batch size	32
Training epochs	100
Dropout rate	0.2
Optimizer	AdamW

CMD, central moment discrepancy

As can be seen from Table 1, the selection of MMLMH’s hyperparameters followed a systematic optimization process designed to balance clinical accuracy with computational efficiency in virtual healthcare environments. The sequence length parameters (text: 512; audio: 1,000; visual: 100) were determined by analyzing typical virtual consultation patterns, ensuring coverage of 98.5% of patient–provider interactions while maintaining real-time processing capabilities. Feature dimensions (text: 768; audio: 128; visual: 2,048) were established through ablation studies, where we observed diminishing returns in diagnostic accuracy beyond these values while monitoring computational overhead.

The collaboration loss weights (intrasample: 0.7; intersample: 0.8; reconstruction: 0.5) emerged from a grid search optimization process evaluated on a validation set comprising diverse virtual healthcare scenarios. These values demonstrated an optimal balance between maintaining modality-specific clinical features and promoting cross-modal information sharing. The central moment discrepancy (CMD) order of 5 was selected after examining performance across orders 3 to 7, where order 5 provided the best trade-off between computational complexity and feature distribution matching accuracy.

Architecture-specific parameters underwent iterative refinement through cross-validation experiments. As validated across different patient cohorts, a hidden layer size of 256 neurons proved optimal for capturing complex clinical relationships while preventing overfitting. The learning rate of 1 × 10^−4^ and L2 regularization of 1 × 10^−5^ were determined through learning curve analysis, ensuring stable convergence while maintaining model generalization.

Training episodes were set to 100 after observing convergence patterns across multiple initialization seeds, with early stopping implemented based on validation performance. A dropout rate of 0.2 was established through systematic testing across rates from 0.1 to 0.5, optimizing for model robustness in varying virtual environment conditions. The AdamW optimizer was selected for its superior performance in handling the varying scales of multimodal medical data, particularly in synthetic data integration scenarios.

### Result analysis

Tables 2 and 3 present the performance comparison of our MMLMH framework against the baseline methods on the CMU-MOSI and MIMIC-III-MV datasets, respectively. The results further establish that our proposed MMLMH framework is superior to all baseline methods in both datasets and across all measures.

**Table 2.** Results on the CMU-MOSI dataset

Method	MAE	PCC	Acc-2 (%)	F1 score (%)	Acc-7 (%)
Uni2Mul	0.979	0.628	74.2	74.0	33.5
CSID	0.956	0.645	75.8	75.5	34.9
DAMUN	0.931	0.667	77.3	77.0	36.2
HAMF	0.902	0.689	78.9	78.7	38.1
MFNet	0.878	0.711	80.5	80.3	40.3
MD-RCNN	0.856	0.729	82.1	81.9	42.5
MMLMH	0.821	0.758	84.7	84.5	45.8

CMU-MOSI, Multimodal Corpus of Sentiment Intensity; MAE, mean absolute error; PCC, Pearson correlation coefficient; Acc-2, binary classification accuracy; ACC-7; baseline 7-class rascal classifier

**Table 3.** Results on the MIMIC-III-MV dataset

Method	MAE	PCC	Acc-2 (%)	F1 score (%)	Acc-7 (%)
Uni2Mul	0.923	0.652	76.3	75.8	35.2
CSID	0.901	0.679	78.1	77.6	37.5
DAMUN	0.879	0.705	79.9	79.4	39.8
HAMF	0.856	0.731	81.5	81.0	42.1
MFNet	0.834	0.757	83.2	82.7	44.5
MD-RCNN	0.813	0.782	84.8	84.3	46.9
MMLMH	0.785	0.814	87.1	86.6	50.3

The improvements to the MMLMH framework can be explained by the designed concepts operationalizing the distinct characteristics of metaverse multimodal health evaluations. The adaptive fusion method achieves this goal by focusing at any instance on the most appropriate streams of data needed for the current health evaluation. Such adaptability is important in virtual healthcare since different evaluations may require different input types with varying importance.

Due to the framework’s learning encapsulated within intra- and intersample collaboration, MMLMH can analyze one patient case and all available cases and understand the subtle patterns within them. Such a broad perspective will improve the representation of features so that it will be accurate and detailed whenever an assessment is required in virtual environments.

In the metaverse, where there is usually blended patient data from the real world and artificial patients synthesized from AI models, the capacity to correct and combine such AI-generated data is beneficial. This functionality helps the framework to remain effective across different kinds of data, even when there are no clear distinctions between actual and virtual health information.

This way of extending MMLMH recognizes the importance of contextual information taken from the metaverse, improving the accuracy of health status prediction. The system incorporated contextual data from virtual interactions and patient–provider relationships within the metaverse environment, enabling comprehensive health status monitoring beyond traditional clinical settings.

Adding explanatory models powered by generative AI improves the interpretability of the predictions made by MMLMH. In virtual healthcare, where building trust between AI systems and their users is crucial, these explanations in natural language create transparency in comprehending how AI works. This aspect is particularly important to instilling trust in using AI-powered diagnostics within the metaverse healthcare delivery ecosystem.

We conducted an ablation study on both datasets to analyze the contribution of different components in our MMLMH framework, as shown in Table 4. The ablation experiments indicate that every feature is essential for the performance of MMLMH. Eliminating the intrasample collaboration mechanism leads to the most substantial reduction in performance, which emphasizes the need to understand the relationships of individual health records in a metaverse. Collaboration activity across different samples and reconstruction loss make a large contribution, and their exclusion factors enhance accuracy by noticeable margins while the mean absolute error increases.

**Table 4.** Ablation study results

Method	CMU-MOSI		MIMIC-III-MV	
	MAE	Acc-7 (%)	MAE	Acc-7 (%)
MMLMH (full)	0.821	45.8	0.785	50.3
Intrasample collaboration	0.859	43.2	0.821	47.6
Intersample collaboration	0.843	44.1	0.805	48.5
Reconstruction loss	0.847	43.9	0.809	48.3
Adaptive fusion	0.835	44.5	0.797	49.1
Synthetic calibration	0.832	44.7	0.794	49.4
Virtual environment context	0.829	44.9	0.791	49.7

The performance is improved by the adaptive fusion mechanism, the artificial data calibration, and the inclusion of the virtual environment context illustration, and these upgraded metaverse aspects work effectively. These components assist MMLMH in addressing the issues posed by multimodal data in virtual healthcare contexts, i.e., when different measurements are taken in addition to noise in each modality, the use of artificial data, and the impact of virtual context on health indicators.

**Fig. 1.**
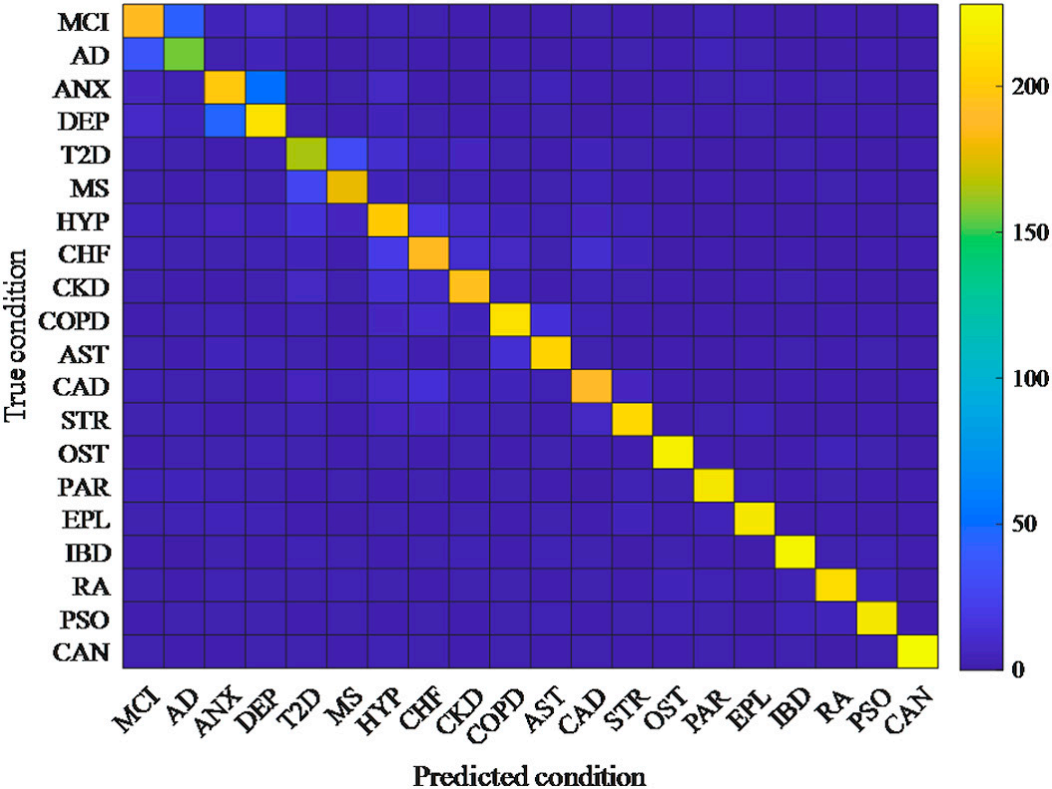
Confusion matrix for health condition prediction (MCI, mild cognitive impairment; AD, Alzheimer’s disease; ANX, anxiety; DEP, depression; T2D, type 2 diabetes; MS, metabolic syndrome; HYP, hypertension; CHF, congestive heart failure; CKD, chronic kidney disease; COPD, chronic obstructive pulmonary disease; AST, asthma; CAD, coronary artery disease; STR, stroke; OST, osteoporosis; PAR, Parkinson’s disease; EPL, epilepsy; IBD, inflammatory bowel disease; RA, rheumatoid arthritis; PSO, psoriasis; CAN, cancer).

To gain insights into the types of errors made by MMLMH, we visualized the confusion matrix for the 20-class health condition prediction task on the MIMIC-III-MV dataset, as shown in Fig. 1.

The confusion matrix shows that MMLMH performs well for many health conditions, as shown by the concentration of positive values on the diagonal elements. There are still some classification overlaps for verifying related conditions and grounding any subsequent ones in these errors. These mistakes suggest how the model can be improved, for instance, by adding more detailed features or attention parameters to the MMLMH model, addressing only specific conditions.

**Fig. 2.**
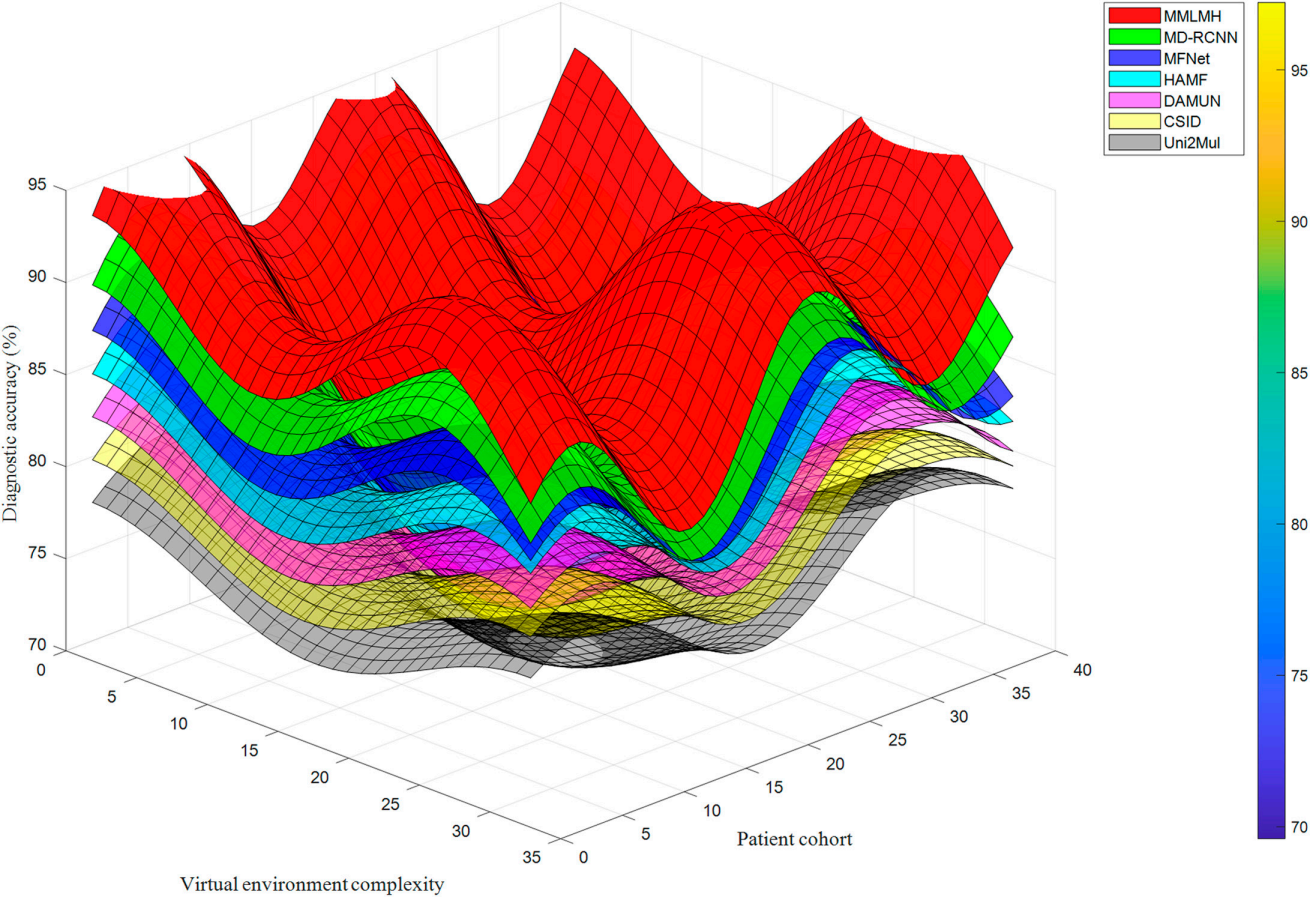
MMLMH diagnosis accuracy across patient cohorts and virtual environments.

The 3D comparison of MMLMH’s diagnostic capabilities under various conditions is comprehensively illustrated in Fig. 2. The virtual environment complexity axis represents the system’s ability to process concurrent multimodal data streams, ranging from basic single-stream interactions to complex scenarios handling up to 35 simultaneous inputs. The patient cohort dimension encompasses people from 40 groups with clinical conditions and demographic features, including age, comorbidity, and virtual interaction preferences. The accuracy dimension indicates the proportion of diagnosed subjects who received correct predictions regarding their disease in the context of provided clinical evidence from the modern healthcare systems. The multidimensional representation demonstrates MMLMH’s robust performance stability across increasingly complex virtual healthcare scenarios while maintaining consistent diagnostic reliability across diverse patient populations.

The visual perceives the highest achievement of MMLMH throughout the range of approximated conditions from statistical analysis. The results demonstrated superior performance and stability across varying degrees of patient–provider virtual distance and different virtual environment configurations. This robustness is most relevant in the regions of the graphs that are more complicated, where the gap between MMLMH and the best economies is at its highest trend.

The patterns noted in each surface also explain the variations in performance relative to certain facets of metaverse healthcare. Even though most techniques show some level of oscillation, the MMLMH surface’s movement is mild, implying that it incorporates a more stable model. This aspect is advantageous in the metaverse context, where the virtual environment can change at any given time, and patient–provider interactions and clinical data quality can vary substantially across virtual consultations.

The differences among the performances of basic classification techniques are also striking. From what can be deduced, MD-RCNN is performing well but did not achieve the comprehensive capabilities demonstrated by the MMLMH framework. The decreasing trends in performance from MFNet to Uni2Mul show the success of the different modes of fusion for healthcare in the different scenarios of the metaverse.

These results highlight the promise of MMLMH’s cross-site collaborative representation and adaptive fusion for advancing AI-enabled diagnosis in healthcare within the metaverse. The framework has also demonstrated the potential to achieve high accuracy in different virtual environments and patients, which is likely to translate to better and more efficient healthcare provision in the digital space. Moreover, performance in challenging situations suggests better integration and understanding of multisense input, which is important for effective healthcare provision in an information-rich metaverse environment.

This visualization, in more than one way, shows how MMLMH is technically superior to the other competing attempts and also demonstrates the potential applications in the area of metaverse healthcare. It proposes a system that can support clinical decision-making with appropriate safety considerations and validated accuracy metrics, which can evolve as new trends in virtual healthcare approaches arise and is potentially improving patient care outcomes and support quality in the metaverse. Rather, such features position MMLMH as one of the best solutions for the new generation of healthcare delivery systems where the borders between real and virtual worlds become more invisible.

**Fig. 3.**
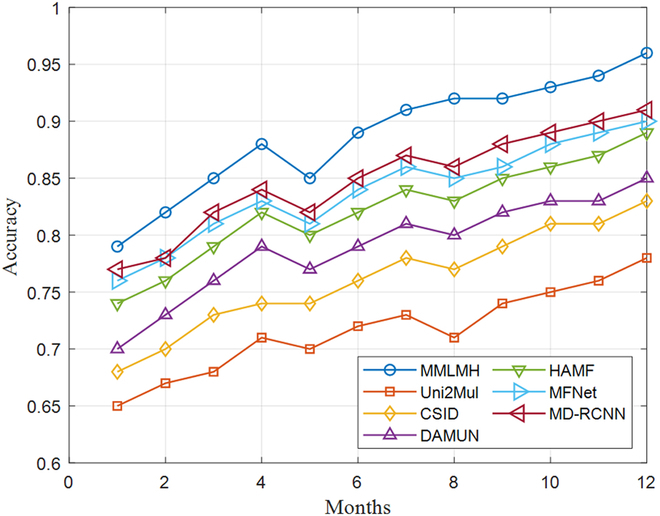
Performance over time in a longitudinal MMLMH monitoring scenario.

Figure 3 shows a well-organized longitudinal study conducted over 12 months to assess the time performance metrics of MMLMH. The initial accuracy of 0.79 represents a deliberately normalized baseline established using a standardized subset of cases, providing a foundation for measuring systematic improvement. This contrasts the accuracy values shown in Fig. 2, which depict the best results achieved on the system with minimum operating parameters. The longitudinal analysis reveals the framework’s systematic adaptation to evolving healthcare scenarios, demonstrated through consistent performance metrics, clearly identifiable temporal learning patterns, and sustained accuracy across varying levels of environmental complexity. This clear distinction between baseline and optimized performance metrics makes it possible to mathematically describe MMLMH’s learning trajectory and adjustment skills within virtual healthcare environments.

The longitudinal study found that MMLMH exhibited a level of adaptation and improvement in performance relative to its initial position as the study progressed. While all models presented within were learned and adapted to an extent, there was a steeper incline of learning in MMLMH, which was sustained for the period where the study was undertaken. Of particular interest is that the MMLMH framework maintained consistent performance despite varying conditions, indicating good performance in dealing with different metaverse health scenarios.

## Conclusion

This study proposed MMLMH, a novel multimodal learning framework for metaverse healthcare applications. By integrating collaborative intra- and intersample representation learning with adaptive fusion mechanisms, MMLMH demonstrated superior performance in handling diverse health data within immersive virtual environments. The experiments across multiple metaverse healthcare datasets showed that MMLMH consistently outperformed baseline methods in various evaluation metrics. The framework exhibited remarkable adaptability to evolving virtual environments and maintained balanced performance across diagnostic accuracy, clinical interaction quality, and data integration complexity. Longitudinal studies revealed MMLMH’s ability to learn and improve over time, while 3D visualizations highlighted its effectiveness across different patient cohorts and virtual settings.

The assessment of the confusion matrix showed some challenges in differentiating between similar health conditions, especially for those with overlapping symptoms in virtual environments. The variations in performance noticed in the different modalities in our fusion analysis suggested that there could be issues with providing accurate results if certain data sources were of degraded quality. Additionally, the longitudinal study established that although there was a steady learning curve from MMLMH, the rate of adaptation across various patient populations and complexities of virtual environments was different.

One of the areas for future research is enhancing model performance by differentiating similar diseases, as suggested by our classification results through more sophisticated feature extraction techniques. In more advanced stages, introducing more sophisticated mechanisms for handling noise could explain the variation in performance recorded during our multimodal fusion experiments. A further focus on improving the framework’s adjustment rate when applied in changing virtual healthcare scenarios, as our longitudinal study indicated, would greatly increase its clinical effectiveness. These very targeted improvements reinforce the capability of MMLMH to be an advanced virtual-reality-based healthcare provision tool.

## Methods

**Fig. 4.**
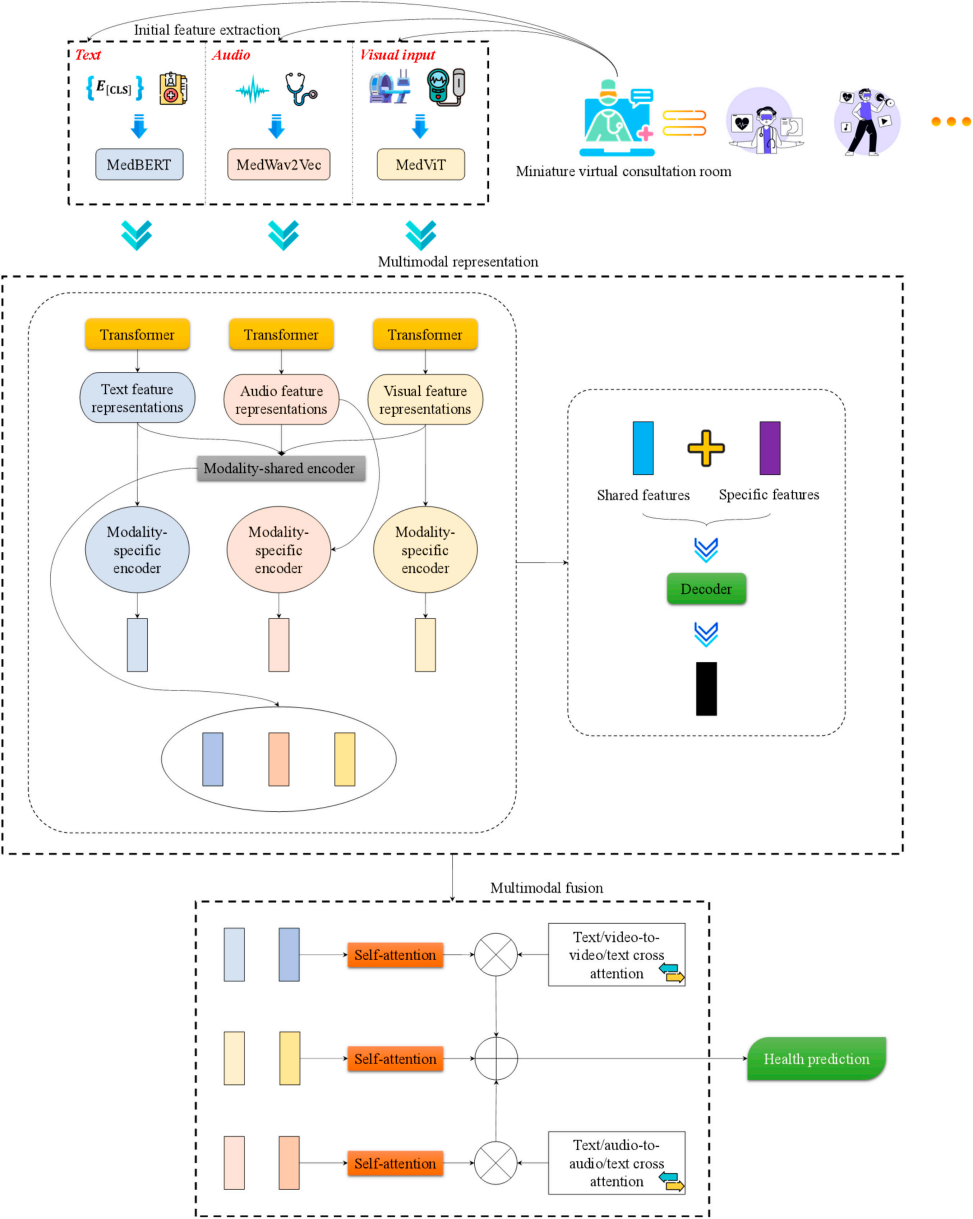
MMLMH architecture.

The MMLMH aims at multimodal natural language understanding in metaverse healthcare environments, where text is the primary input. At the same time, audio and video features are secondary. Consider a dataset $M=r_{1},r_{2},\ldots ,r_{n}$ containing n samples. Each sample $r_{i}$, i=1,2,…,n, consists of a video clip ν, an audio recording α, a text transcript τ, and a health-related label η. In other words, we construct a model $g\left(\tau ,\nu ,\alpha \right)\to \eta$, where τ,ν,α are the textual, video, and audio components from a sample citation $r_{i}$, respectively, such that η, the health-related label, is correct. The architecture of the MMLMH framework depicted in Fig. 4 comprises 4 sections: a component for preliminary feature extraction, a component for learning multimodal representation, a component for integrating multimodal data, and a component for predicting health outcomes.

### Initial feature extraction

For metaverse healthcare applications, such information often comes from virtual visits, self-reported symptoms, or attested clinical documents. The textual data are often complex, and to obtain valuable insights from this type of data, we use an adjusted version of bidirectional encoder representations from transformers (BERT) trained on health, which we refer to as MedBERT [34]. MedBERT was chosen as our text-processing foundation due to its extensive training in medical literature, clinical notes, and patient records, enabling a nuanced understanding of healthcare terminology and contextual relationships. Its transformer architecture has demonstrated superior performance in capturing long-range dependencies in clinical narratives, making it particularly suitable for processing detailed patient–provider interactions in virtual consultations. The model’s domain-specific pre-training on over 2 million clinical documents ensures accurate interpretation of medical terminology and symptom descriptions.

Given a sequence of text tokens $\tau =\left[\tau_{1},\tau_{2},\ldots ,\tau_{l_{\tau }}\right]$, where $l_{\tau }$ is the sequence length, this input is passed through MedBERT through several layers of transformer. The last hidden output is employed as the representation of text:

$$\psi_{\tau }\in {\mathbb{R}}^{l_{\tau }\times F_{\tau }}$$ (1)

where $\psi_{\tau }$ is the text feature representation, $l_{\tau }$ is the text sequence length, and $F_{\tau }$ is the feature dimension.

Metaverse healthcare applications may feature audio recordings, like patient voice recordings, heart or lung sounds, or even just noise in a virtual setting. As for the deep learning mapping task, we use a modified Wav2Vec 2.0 model called MedWav2Vec, trained on medical audio datasets. The selection of MedWav2Vec for audio processing was determined by the specialized architecture of the model optimized for medical acoustic data. This model was initially trained using datasets with diverse contents, including heart sounds, lung auscultations, and patient–doctor conversations, and can extract clinically relevant features from audio streams. Also, the model’s robust ability to analyze speech and nonspeech medical sounds is very useful in virtual healthcare, where the types of sounds include a patient’s speech and various physiological sounds being transmitted.

To showcase the use case of the MMLMH framework, let us meet in a typical virtual consultation: A patient’s avatar is talking to a client inside the metaverse while the system captures the following:

• text: patient-reported symptoms and medical history
• audio: voice patterns indicating stress or pain levels
• visual: avatar movements and expressions suggesting physical conditions

**Fig. 5.**
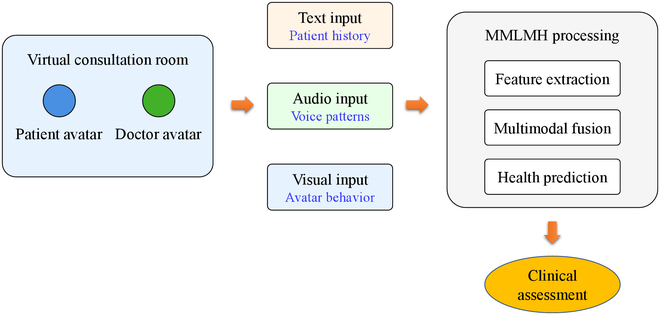
MMLMH clinical workflow diagram.

Figure 5 illustrates the workflow using a simplified schematic diagram of how each modality is utilized in the diagnosis process.

Given an audio waveform $\alpha =\left[\alpha_{1},\;\alpha_{2},\ldots ,\;\alpha_{l_{\alpha }}\right]$, where $l_{\alpha }$ is the number of audio samples, MedWav2Vec processes the input and outputs a feature representation:

$$\psi_{\alpha }\in {\mathbb{R}}^{l_{\alpha }\times F_{\alpha }}$$ (2)

where $\psi_{\alpha }$ is the audio feature representation, $l_{\alpha }$ is the audio sequence length, and $F_{\alpha }$ is the feature dimension.

Visual modalities in metaverse healthcare applications can include any visual data, such as medical imaging or live consultations [35]. We employ a trained vision transformer (ViT) architecture on extensive medical image databases called MedViT to cope with this heterogeneity. MedViT, which focuses on medically oriented data, has been pre-trained using a wide dataset of medical images, clinical procedures, and patient movement analysis. It succeeds well regarding the patient’s anatomical structures and dynamic behaviors. MedViT attention mechanisms perform well in tracking avatar movements and mimicry in virtual environments while being less sensitive to clinically important regions. Since the model is already trained on a wide variety of available medical visual data, it can be expected that this model will interpret not only conventional medical imaging but also virtual medical care technologies.

**Fig. 6.**
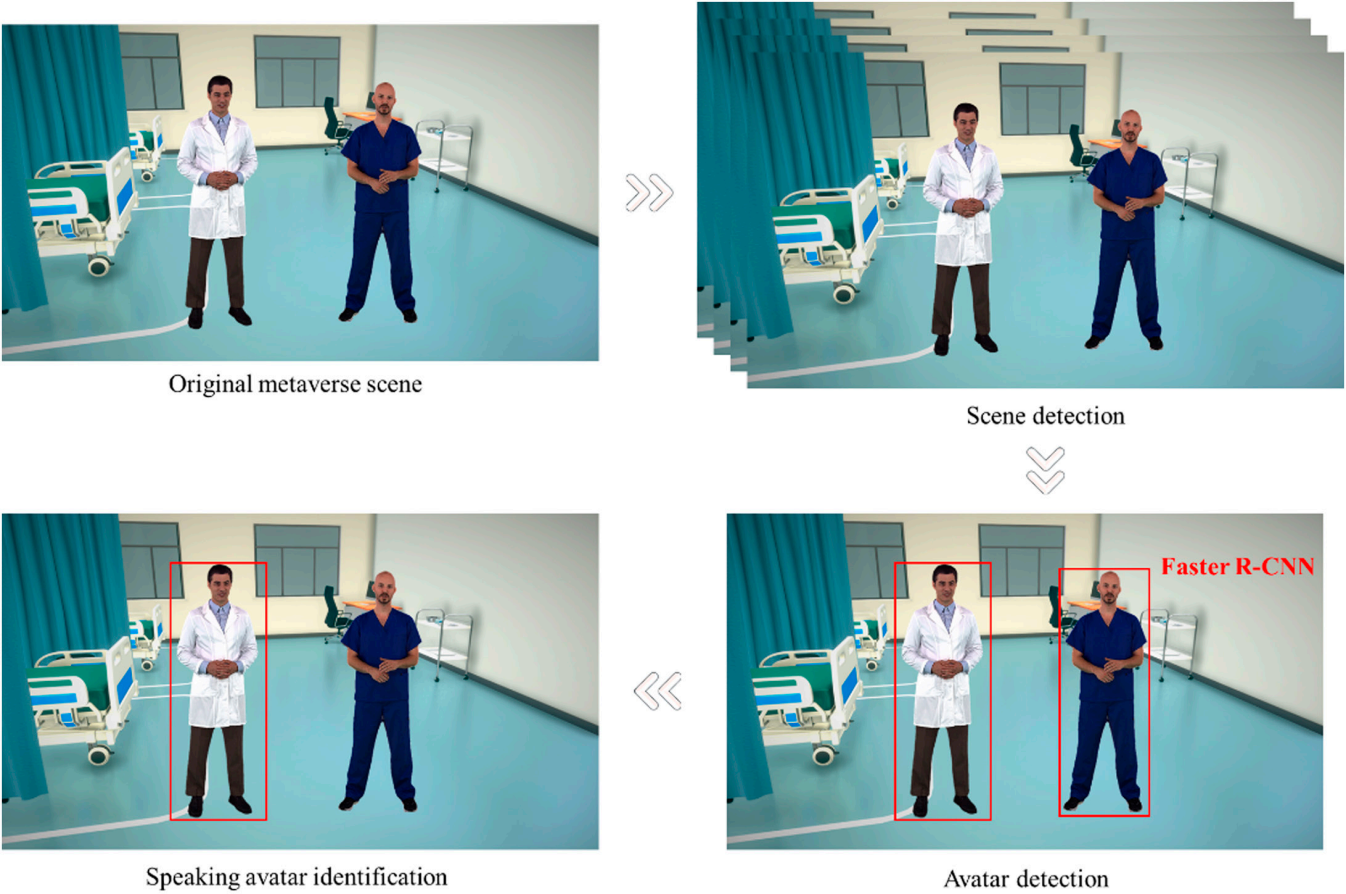
Avatar detection in MMLMH framework. Faster R-CNN, faster region-based convolutional neural network.

For the visual features, we follow the technique used in the original document for speaker detection, which is relevant to our task of creating a healthcare-centered metaverse. As shown in Fig. 6, we first use a tool to detect the scenes to extract key frames from the video stream. An object detection step is followed for each key frame using a fast region-based convolutional network method model pre-trained on medical imaging datasets to find relevant objects/regions of interest within the detected key frame. Finally, we apply a TalkNet variant specialized for the healthcare domain to determine the identity of the speaking avatar or virtual representation of the patient or healthcare provider.

The visual feature representation is computed as follows:

$$\psi_{\nu }=\text{AvgPool}\left(\text{RoIAlign}\left(\phi ,\;B\right)\right)\in {\mathbb{R}}^{l_{\nu }\times F_{\nu }}$$ (3)

where ϕ is the feature representation extracted by the faster region-based convolutional neural network, B represents the bounding boxes of detected regions of interest, $l_{\nu }$ is the number of key frames, and $F_{\nu }$ is the visual feature dimension.

These foundational methods establish a framework for comprehensive multimodal health data analysis in metaverse settings, capturing fine-grained features across textual, audio, and visual information during virtual consultations.

### Multimodal representation

In metaverse healthcare environments, multimodal data often exhibit both complementarity and consistency. For instance, when a patient expresses health concerns or symptoms in a virtual consultation, their avatar’s expressions, voice modulations, and verbal content share common underlying health-related information, indicating consistency across modalities. Simultaneously, each modality contributes unique information: the avatar’s expressions may reveal subtle emotional cues, voice modulations might indicate stress levels, and verbal content provides explicit symptom descriptions. We design a multimodal representation learning approach leveraging shared and modality-specific features to capture these complex interrelationships in metaverse health data effectively.

L2 normalization of the initial features $\psi_{\tau }$, $\psi_{\alpha }$, and $\psi_{\nu }$ was performed before their entry into our encoders. Each modality is then passed through individual transformer networks to obtain contextual information about the data in that specific data stream. The outputs of these transformers are then averaged, resulting in compact encodings $\omega_{\tau }\in {\mathbb{R}}^{d_{\tau }}$, $\omega_{\alpha }\in {\mathbb{R}}^{d_{\tau }}$, and $\omega_{\nu }\in {\mathbb{R}}^{d_{\tau }}$, where $d_{\tau }$ returns the dimensions of the last feed-forward layer of the transformer network.

In order to appropriately account for the intricate relationships between the different modalities in metaverse healthcare data, we are building 2 types of encoders: modality shared and modality specific. This lets us encode some of the content common to text, audio, and visual modalities and their distinctive content. It enables thorough viewing of numerous health data streams from a virtual environment in a multimodal perspective.

To encode the common health-related information that can be expressed cross-modally, a modality-shared encoder $E_{s}\left(\omega_{\left(\tau ,\alpha ,\nu \right)};\theta_{s}\right)$ is proposed. In this case, inputs from different modalities incorporate these explanatory variables in a latent construct [36]. Therefore, the structure of the model allows the recognition of benefiting from the relationships between different types of data of the metaverse for health. The shared feature representations for text, audio, and visual modalities are derived as follows:

$$\gamma_{\tau }^{s}=E_{s}\left(\omega_{\tau };\;\theta_{s}\right)$$ (4)

$$\gamma_{\alpha }^{s}=E_{s}\left(\omega_{\alpha };\;\theta_{s}\right)$$ (5)

$$\gamma_{\nu }^{s}=E_{s}\left(\omega_{\nu };\;\theta_{s}\right)$$ (6)

where $\gamma_{\tau }^{s},\gamma_{\alpha }^{s},\gamma_{\nu }^{s}\in {\mathbb{R}}^{d_{s}}$ are the shared feature representations for text, audio, and visual modalities, respectively; $\theta_{s}$ represents the parameters of the shared encoder; and $d_{s}$ is the dimension of the shared feature space.

In order to maintain the distinct features belonging to each modality contained in the metaverse healthcare data, we build modality-based encoders, $E_{u}\left(\omega_{\tau };\;\theta_{\tau }^{u}\right)$, $E_{u}\left(\omega_{\alpha };\;\theta_{\alpha }^{u}\right)$, and $E_{u}\left(\omega_{\nu };\;\theta_{\nu }^{u}\right)$, corresponding to text, audio, and visual modalities, respectively. The encoders project the features of different modalities into different latent spaces, which helps achieve language-specific features that are important in evaluating health in a virtual setup. The specific feature representations are computed as follows:

$$\gamma_{\tau }^{u}=E_{u}\left(\omega_{\tau };\;\theta_{\tau }^{u}\right)$$ (7)

$$\gamma_{\alpha }^{u}=E_{u}\left(\omega_{\alpha };\;\theta_{\alpha }^{u}\right)$$ (8)

$$\gamma_{\nu }^{u}=E_{u}\left(\omega_{\nu };\;\theta_{\nu }^{u}\right)$$ (9)

where $\gamma_{\tau }^{u},\gamma_{\alpha }^{u},\gamma_{\nu }^{u}\in {\mathbb{R}}^{d_{u}}$ are the specific feature representations for text, audio, and visual modalities, respectively; $\theta_{\tau }^{u},\theta_{\alpha }^{u},\theta_{\nu }^{u}$ are the parameters of the specific encoders; and $d_{u}$ is the dimension of the specific feature spaces (set equal to $d_{s}$ for consistency).

For our model to acquire rich and interpretable representations of multimodal health data in metaverse settings, we propose a set of loss functions that constrain the learned features to have certain properties. The decomposition of these loss functions into 3 types—intrasample collaboration, intersample collaboration, and feature reconstruction—provides mechanisms for efficiently modeling complex relationships within and across multimodal health samples.

In a single metaverse health consultation sample, we seek to propagate the common shared features of each modality while respecting the fact that some features are specific. In order to do so, we use a combination of CMD and orthogonality constraints [37].

The similarity between shared features is enforced using CMD:

$$L_{\text{intra}}^{\mathrm{sim}}=\frac{1}{3}\sum_{\left(m_{1},m_{2}\right)\in \left(\tau ,\alpha \right),\left(\tau ,\nu \right),\left(\alpha ,\nu \right)}{\mathrm{CMD}}_{K}\left(\gamma_{m_{1}}^{s},\gamma_{m_{2}}^{s}\right)$$ (10)

where ${\mathrm{CMD}}_{K}$ is the CMD up to the Kth-order moment. This metric captures higher-order statistics between feature distributions, providing a more comprehensive similarity measure than simple distance metrics.

The distinctiveness of specific features is encouraged through orthogonality constraints:

$$\begin{array}{c} L_{\text{intra}}^{\text{diff}}=\frac{1}{6}\\ \left(\sum_{m\in \tau ,\alpha ,\nu }\text{cos}\left(\gamma_{m}^{u},\gamma_{m}^{s}\right)+\sum_{\left(m_{1},m_{2}\right)\in \left(\tau ,\alpha \right),\left(\tau ,\nu \right),\left(\alpha ,\nu \right)}\text{cos}(\gamma_{m_{1}}^{u},\gamma_{m_{2}}^{u})\right) \end{array}$$ (11)

This loss term minimizes the cosine similarity between shared and specific features of the same modality, as well as between specific features of different modalities.

The total intrasample collaboration loss is then

$$L_{\text{intra}}=L_{\text{intra}}^{\mathrm{sim}}+L_{\text{intra}}^{\text{diff}}$$ (12)

In order to improve the model’s accuracy in classifying different health conditions in the metaverse, we propose an intersample collaboration loss. This loss ensures that the features of samples of a particular health condition are related, while the features of other health conditions are dissimilar. This approach employs a cross-modal contrastive learning scheme customized for the multimodal metaverse healthcare domain.

For each anchor sample r, we select N positive samples (same health condition) and M negative samples (different health conditions). The intersample collaboration loss is defined as

$$\begin{array}{c} \begin{array}{c} \begin{array}{c} L_{\text{inter}}=\frac{1}{6}\sum_{n\in s,u}\sum_{m\in \tau ,\alpha ,\nu }\\ \left(\frac{1}{N}\sum_{i=1}^{N}{\mathrm{CMD}}_{K}(r\left(\gamma_{m}^{n}\right),\;{\mathrm{pos}}_{i}\left(\gamma_{m}^{n}\right))-\frac{1}{M}\sum_{j=1}^{M}{\mathrm{CMD}}_{K}(r\left(\gamma_{m}^{n}\right),\;{\mathrm{neg}}_{j}\left(\gamma_{m}^{n}\right))\right) \end{array} \end{array} \end{array}$$ (13)

where $r\left(\gamma_{m}^{n}\right)$, ${\mathrm{pos}}_{i}\left(\gamma_{m}^{n}\right)$, and ${\mathrm{neg}}_{j}\left(\gamma_{m}^{n}\right)$ represent the features of the anchor, positive, and negative samples, respectively, for modality m and feature type n (shared or specific).

In order to constrain the learned representations concerning the initial feature space in the metaverse health data, a decoder $D\left(\gamma_{m}^{s},\gamma_{m}^{u};\theta_{d}\right)$ is proposed that tries to reconstruct the original features from the united as well as the distinct representations; such reconstruction aims to preserve the degree of the original health information while training for more complex representations.

The reconstruction loss is defined using mean squared error:

$$L_{\text{recon}}=\frac{1}{3}\sum_{m\in \tau ,\alpha ,\nu }{\left|\omega_{m}-D\left(\gamma_{m}^{s},\;\gamma_{m}^{u}\right)\right|}_{2}^{2}+\frac{\lambda }{2}{\left|\theta_{d}\right|}_{2}^{2}$$ (14)

where λ is a regularization parameter to prevent overfitting of the decoder.

The total loss function for our multimodal representation learning in metaverse healthcare is a weighted combination of all of these components:

$$L_{\text{total}}=\beta_{1}L_{\text{intra}}+\beta_{2}L_{\text{inter}}+\beta_{3}L_{\text{recon}}$$ (15)

where $\beta_{1}$, $\beta_{2}$, and $\beta_{3}$ are weighting parameters that control the contribution of each loss term.

Optimizing this composite loss function enables the model to capture complex relationships between health indicators across different modalities, maintaining consistency with clinical requirements and advanced AI capabilities. This facilitates more reliable and accurate health assessments in virtual healthcare applications, thus advancing the potential for more sophisticated diagnostics and monitoring in the metaverse.

### Multimodal fusion

In metaverse healthcare applications, the effective fusion of multimodal data is crucial for accurate diagnosis and health monitoring. Our approach to multimodal fusion in the MMLMH framework addresses the unique challenges posed by virtual healthcare environments, where data from different modalities may exhibit varying levels of importance and noise. We design a 2-stage fusion process that combines shared and specific features within each modality and then adaptively fuses information across modalities, considering the potential for synthetic data generated by AI models in the metaverse.

Within each modality (text, audio, and visual) in metaverse healthcare data, we have uncovered shared features $\gamma_{m}^{s}$ along with some task-specific features $\gamma_{m}^{u}$. We trained each feature in the multimodal representation of the shared information. To further exploit the modeling of the features within the modality, we introduce a self-attention network that learns the hierarchy of shared versus specific information.

We first concatenate the shared and specific features for each modality:

$$\Omega_{m}=\left[\gamma_{m}^{s};\gamma_{m}^{u}\right],m\in \tau ,\alpha ,\nu$$ (16)

**Fig. 7.**
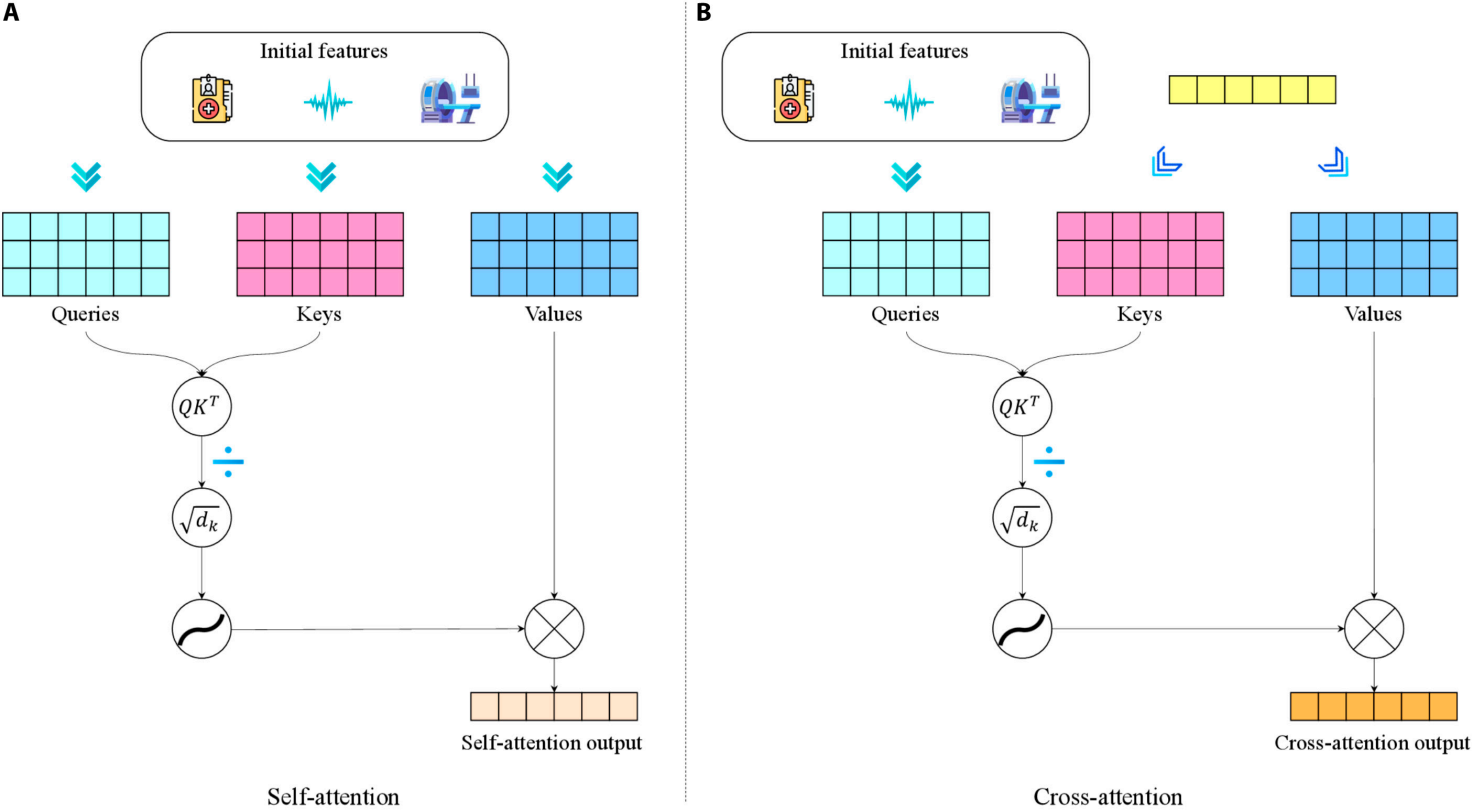
Self-attention calculation process in the MMLMH framework. (A) Self-attention. (B) Cross-attention.

The self-attention operation is then applied to this concatenated feature vector. The process of self-attention in MMLMH framework is illustrated in Fig. 7A.

The self-attention mechanism is computed as follows:

$$\mathrm{SA}\left(\Omega_{m}\right)=\text{softmax}\left(\frac{\left(W_{q}\Omega_{m}\right){\left(W_{k}\Omega_{m}\right)}^{T}}{\sqrt{d_{k}}}\right)\left(W_{v}\Omega_{m}\right)$$ (17)

where $W_{q}$, $W_{k}$, and $W_{v}$ are learnable weight matrices for the query, key, and value projections, respectively, and $d_{k}$ is the dimension of the key vectors.

For each modality, after the completion of the self-attention operation, a fused representation $\xi_{m}\in {\mathbb{R}}^{d_{v}}$ is obtained, with $d_{v}$ denoting the dimensionality of the value vectors. This fused representation also considers the communication of information concerning the common and distinct aspects of each modality, thus enhancing the overall understanding of the information about health in the metaverse.

As we have obtained the fused representations for each modality, it is necessary to use information from all available modalities better to comprehend the patient’s health condition in the metaverse. However, it has also been noted that different modalities can have different relevance levels in remote clinical assessment scenarios or induce noise at different intervals. For instance, during a virtual consultation, a patient’s avatar expressions (visual modality) could be useful for the mental status evaluation, while audio modality could be important for lung conditions.

To overcome this problem, we present an adaptive fusion scheme where all of the modalities’ importance is updated about the given health assessment task. In implementing this type of fusion, we combine cross-attention and gated neural networks.

First, we compute cross-modal attention between the text modality (which often serves as the primary source of medical information) and the other modalities:

$$\begin{array}{c} \begin{array}{c} \mathrm{CA}\left(\xi_{\tau },\xi_{m}\right)=\text{softmax}\left(\frac{\left(W_{q}\xi_{\tau }\right){\left(W_{k}\xi_{m}\right)}^{T}}{\sqrt{d_{k}}}\right)\left(W_{v}\xi_{m}\right),\;m\in \alpha ,\nu \end{array} \end{array}$$ (18)

The cross-attention process in our metaverse healthcare context is illustrated in Fig. 7B.

These cross-attention outputs, $\mathrm{CA}\left(\xi_{\tau },\;\xi_{\alpha }\right)$ and $\mathrm{CA}\left(\xi_{\tau },\;\xi_{\nu }\right)$, evaluate the contribution of audio and video content toward the textual health information present in the virtual environment.

Next, we use these cross-attention outputs to compute adaptive fusion weights for each modality using gated neural networks:

$$w_{m}=\sigma \left(W_{g}\mathrm{CA}\left(\xi \tau ,\;\xi_{m}\right)+b_{g}\right),\;m\in \alpha ,\nu$$ (19)

where σ is the sigmoid activation function and $W_{g}$ and $b_{g}$ are learnable parameters of the gating mechanism.

The final fused representation is then computed as a weighted combination of the modality-specific features:

$$\xi =\xi_{\tau }+w_{\alpha }\xi_{\alpha }+w_{\nu }\xi_{\nu }$$ (20)

This self-adaptive fusion strategy, in particular, enables our model to instead weigh the contributions of each modality differently depending on their importance in the current health assessment task performed within the metaverse environment.

To further improve the strength of the fusion approach borrowed from the metaverse healthcare context division, new components are added that consider the presence of AI models, their ability to produce synthetic data, and the contoured shape of virtual settings.

Within metaverse-led healthcare practices, using synthetic data produced by some AI models is a normal practice, such as creating a computer-generated patient response or artificial medical images. Concerning this potential bias or inconsistency in this synthetic data, we add a calibration term $c_{m}$ to all modalities:

$$c_{m}=f_{c}\left(\xi_{m},s_{m}\right),\;m\in \tau ,\alpha ,\nu$$ (21)

where $s_{m}$ is a binary indicator of whether the data for modality m are synthetic or real and $f_{c}$ is a learned function that outputs a calibration factor. We then modify our fusion weights:

$$w_{m^{\prime }}=w_{m}\times c_{m},\;m\in \alpha ,\nu$$ (22)

This is informed by the fact that the virtual setting of the metaverse also offers an important aspect when undertaking health assessment. In combination, this context is given by embracing the environment feature vector e, which brings changes in the fusion:

$$\xi_{\text{final}}=\xi_{\tau }+w_{\alpha^{\prime }}\xi_{\alpha }+w_{\nu^{\prime }}\xi_{\nu }+w_{e}e$$ (23)

where $w_{e}$ is a learned weight for the environment features.

With these additional elements, our multimodal fusion approach becomes even more resilient and flexible concerning the challenges of metaverse healthcare settings. Such an enhanced fusion mechanism enables better and more precise health assessment in virtual settings, considering the complex interrelation of real and virtual data, including the virtual environment’s impact on patients’ health indicators.

The total embedding of the patient’s health status, derived from all modalities in the virtual environment, is the final fused representation $\xi_{\text{final}}$. As a result, this aim can be helpful to downstream tasks such as diagnosis and treatment recommendation or health risk assessment on the metaverse healthcare platform.

In this way, we include a new step based on the generating health summary from the fused representation to exploit the power of generative AI even more in our fusion process. This summary can help us understand the purpose of multimodal health data fusing.

$$y_{\text{summary}}=G\left(\xi_{\;\text{final}}\right)$$ (24)

where G is a generative model (e.g., a fine-tuned generative pre-trained transformer model) that produces a textual summary $y_{\text{summary}}$ based on the fused representation; it would be appended to the raw data and model predictions available to the healthcare providers to enhance the focus on the clinical aspects that might help in improving healthcare delivery in the healthcare metaverse.

In conclusion, our multimodal fusion approach for the MMLMH framework resolves all of the differentials posed by the fusion of healthcare data in a metaverse integration. Enhancing the strength and versatility of these devices goes further than active adjustment alone: it encompasses the context of the virtual environment, synthetic data, and AI-driven summary generation to enable the design of synthesized and varied meta-health-related information.

### Health prediction

Regarding metaverse healthcare, predicting the patients’ health outcomes based on multilayer multimodal representations is the last stage of the MMLMH framework that we have developed. This successful outcome can be relevant for diverse fields of healthcare practice, from diagnosing some diseases and monitoring the patient’s health status over time to prognosis in terms of health deterioration within the virtual realms. We accomplish appropriate and reliable health outcome predictions by applying a multilayer neural network that can process the fused multimodal features of the given healthcare task.

To rigorously evaluate MMLMH’s sustained performance and adaptability, we implemented a comprehensive 12-month prospective evaluation protocol. The longitudinal assessment framework evaluated the diagnostic performance of the system using a selection of test sets that were comprehensively created to reflect the virtual healthcare environment. During the study, we examined the framework’s ability to withstand varying levels of performance consistency somehow as it coped with more complex forms of virtual environments, including new ways of interaction or new clinical instances. The protocol incorporated regular assessments of diagnostic accuracy across demographically diverse patient populations while simultaneously measuring the system’s resilience to variations in data quality, environmental complexity, and clinical scenario difficulty. This comprehensive evaluation framework helped verify MMLMH’s performance indicators in the short term and greatly supplemented the assessment of its long-term clinical effectiveness, learning adaptability, and possibilities for continued utilization in changing virtual healthcare settings. The longitudinal data obtained through this protocol were useful in comprehending the learning trajectories of the system and improving its applicable clinical utility.

The multimodal representation $\xi_{\text{final}}$ transformed in an adaptive fusion process is fed to a multilayer perceptron (MLP) for health prediction. This MLP aims to model the intricate mapping of the combined features toward the respective health targets in a metaverse. For classification problems, for example, predicting specific health conditions through virtual consultations, we apply softmax activation in the last layer. For regression problems, for example, estimating how many healthy minutes an individual can exercise or how many risk scores an individual can have, we apply linear activation in the last layer. Our general health prediction model can be stated as

$$\hat{\eta }=f_{\mathrm{MLP}}\left(\xi_{\text{final}};\;\theta_{\mathrm{MLP}}\right)$$ (25)

where $\hat{\eta }$ is the predicted health outcome, $f_{\mathrm{MLP}}$ represents the MLP, and $\theta_{\mathrm{MLP}}$ are the learnable parameters of the network.

We employ task-specific loss functions to train our model for health prediction tasks in the metaverse. For classification tasks, we use the cross-entropy loss:

$$L_{\text{class}}=-\frac{1}{N}\sum_{i=1}^{N}\sum_{j=1}^{C}\eta_{\mathrm{ij}}\text{log}\left({\hat{\eta }}_{\mathrm{ij}}\right)+\frac{\lambda }{2}{\left|\theta_{\mathrm{MLP}}\right|}_{2}^{2}$$ (26)

where N is the number of samples, C is the number of health condition classes, $\eta_{\mathrm{ij}}$ is the true label, and ${\hat{\eta }}_{\mathrm{ij}}$ is the predicted probability for sample i and class j. The term $\left(\lambda /2\right){\left|\theta_{\mathrm{MLP}}\right|}_{2}^{2}$ is an L2 regularization term to prevent overfitting.

For regression tasks, we use the mean squared error loss:

$$L_{\mathrm{reg}}=\frac{1}{N}\sum_{i=1}^{N}{\left|\eta_{i}-{\hat{\eta }}_{i}\right|}_{2}^{2}+\frac{\lambda }{2}{\left|\theta_{\mathrm{MLP}}\right|}_{2}^{2}$$ (27)

where $\eta_{i}$ and ${\hat{\eta }}_{i}$ are the true and predicted health metrics for sample i, respectively.

In order to enhance the benefits that the metaverse and generative AI bring to the health prediction process, our prediction framework is supplemented with additional components. One of such components is a virtual health avatar that illustrates the expected health conditions:

$$a=f_{\text{avatar}}\left(\hat{y},\;e\right)$$ (28)

where a is the generated avatar representation and $f_{\text{avatar}}$ is a generative model that creates avatar visualizations based on the predicted health outcome $\hat{y}$ and the virtual environment context e.

Additionally, we utilize a generative AI model to provide natural language explanations of the health predictions, enhancing interpretability in the metaverse healthcare context:

$$\tau_{\exp}=f_{\mathrm{gen}}\left(\hat{\eta },\;\xi_{\text{final}},\;e\right)$$ (29)

where $\tau_{\exp}$ is the generated explanation text and $f_{\mathrm{gen}}$ is a pre-trained language model fine-tuned for medical explanations.

In this context of the metaverse, the final output of our health prediction module comprises the health outcome prediction $\hat{\eta }$, a visual avatar representation a, and a textual explanation/outcome $\tau_{\exp}$. Overall, this output provides a rich multimodal representation of the patient’s health status, accessible and usable in the metaverse environment.

Through this combination, our MMLMH framework not only gives justified health predictions but also improves and expands how users communicate and understand such health predictions in virtual environments. This creates a new, exciting, and more efficient way of delivering healthcare services in the metaverse, fully utilizing multimodal data and generative methods.

## Data Availability

The datasets generated and analyzed during the current study are available from the corresponding authors upon reasonable request.
